# Free Antiretrovirals Must Not Be Restricted Only to Treatment-Naive Patients: Experience in Uganda suggests that restricting access is not the way forward

**DOI:** 10.1371/journal.pmed.0020276

**Published:** 2005-09-13

**Authors:** Robert Colebunders, Moses Kamya, Fred Semitala, Barbara Castelnuovo, Elly Katabira, Keith McAdam

## Abstract

In some antiretroviral drug distribution programmes, free drugs are provided only, or preferentially, to patients who are treatment-naive. Colebunders and colleagues argue that such a restricted policy is highly problematic.

Despite the “3 by 5” initiative [1], which aims to treat 3 million people with antiretrovirals (ARVs) by the end of 2005, access to ARVs in resource-poor settings remains limited. Moreover, in some ARV-access programmes, free ARVs are provided only, or preferentially, to patients who are ARV naive.

Treatment restricted to patients who are ARV naive was initially the case in Uganda, with the free ARVs provided by the Multi-Country AIDS Programme and the Presidential Emergency Plan for AIDS Relief. While in the policy documents of both projects it was not stated that free ARVs should be given preferentially to ARV-naive patients, many physicians involved in the roll-out of ARVs in Uganda felt that this was what these projects were recommending. Indeed, the Ministry of Health's “Antiretroviral Treatment Policy for Uganda” stated that “those who are clinically eligible and can afford to pay for ART will be encouraged to do so. Those already in privately provided and privately paid ART should be encouraged to remain in this situation. Others who become clinically eligible over time and have the ability to pay or have a third party able to pay in their place or cost share with them, should pay full cost for ART, whether they avail themselves of treatment provided in the public or private sector” [2]. These policy statements were interpreted by many clinicians to mean that patients who are already paying for their drugs can afford to do so and are not a priority for free drugs.

In this essay, we explain the rationale for restricting free access to treatment-naive patients, and then we outline the reasons why such restriction is highly problematic.[Fig pmed-0020276-g002]

**Figure pmed-0020276-g002:**
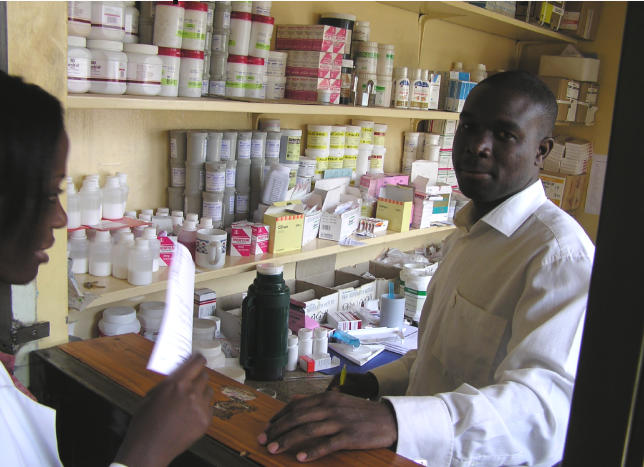
The pharmacy at the Infectious Diseases Institute (Photo: R. Colebunders)

**Figure:**
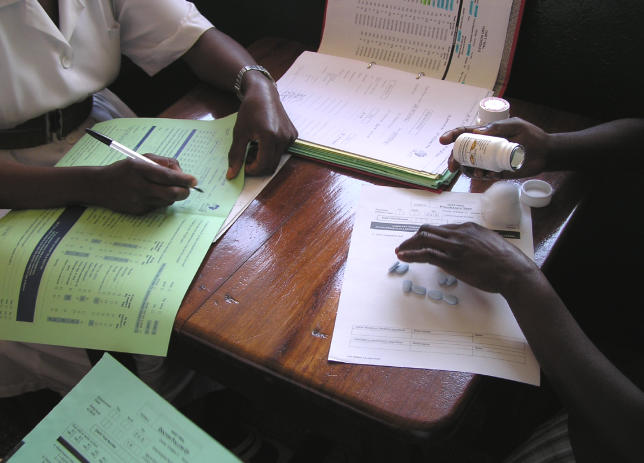
Counting pills during a clinical trial at the Infectious Disease Institute, where the authors are based (Photo: R. Colebunders)

## Why Programmes Restrict Access

There are four principal reasons why free ARVs are being restricted to treatment-naive patients. Firstly, ARV treatment regimens are more predictable in ARV-naive patients [3]. Programmes limited to this population will provide what donors most desire: good outcomes. Secondly, ARV-naive patients will usually respond readily to less expensive first-line ARV regimens, and only a few will require more complex and costly second-line ARVs. Thirdly, results of programmes are easier to compare, because similar ARV regimens are started in more homogeneous, comparable populations. And finally, it is often believed that patients who in the past have been able to buy ARVs should continue to do so.

## Problems with a Restricted-Access Strategy

There are, however, serious problems with this strategy of restricted access. To start, patients are quick to share information, and rumours spread fast regarding ways to obtain free ARVs. When patients learn that ARVs are being given at no cost only to treatment-naive patients, they may not disclose that they have taken ARVs in the past, even to experienced counsellors. We discovered such patients in our centre when they were found to have an undetectable viral load or a CD4+ lymphocyte count higher than expected, when tested prior to starting ARVs. Only when confronted about these results did they acknowledge prior ARV experience. Such withholding of information may result in clinicians choosing inappropriate ARV regimens, thereby placing patients at risk of adverse effects or of development of resistance.[Fig pmed-0020276-g002]

Second, forcing some patients to pay for ARVs can have disastrous consequences for adherence to long-term treatment. Our experience in Uganda has shown that if ARVs are provided for free through an international organisation or a clinical trial, patients achieve excellent adherence. In contrast, if patients or their relatives pay for drugs themselves, treatment failure and the development of resistance is frequently observed [4]. A study in Senegal found that user fees had a negative effect on adherence and were associated with frequent interruptions in treatment [5], and there have been similar findings in Kenya [6], Botswana [7], Cote d'Ivoire [8], and Nigeria [9]. Data from West Africa showed that the level of adherence in the treated population was inversely proportional to the amount of co-payment [10].

In Uganda, currently about 56,000 patients are being treated with ARVs, and about 52% of these treatment regimens are paid for by patients or their relatives (E. Namagala [Uganda Ministry of Health], personal communication). Most of these patients are relatively poor and most have had life-threatening HIV complications. Almost all are struggling with the expense. Many in the near future will be forced, from lack of resources, to either stop their ARVs or take them irregularly. Patients and their families are more willing to sacrifice scarce resources to pay for ARVs when patients are ill, but once they achieve a better health status, additional priorities, such as school fees, tend to take precedence, resulting in poor adherence. It is, therefore, essential that free ARVs be offered to all patients if they are to remain well and if development of resistance is to be thwarted.

Selecting only treatment-naive patients for free ARVs raises a human rights issue.

Third, programmes that offer ARVs only to ARV-naive patients may be relatively slow to enrol patients. All patients beginning ARV therapy for the first time must be carefully selected and prepared, and this can take several clinic visits, including counselling sessions. Moreover, once patients have been started on ARVs, they must be followed closely for side effects and to ensure adherence to the regimen. In contrast, patients already taking ARVs and known to be adherent to their regimen could be enrolled quickly, with less effort.

## The Human Rights Dimension

Finally, we also believe that selecting only ARV-naive patients for free ARVs raises a human rights issue. Can treatment be denied to those who have somehow found money to initiate therapy—often forestalling their demise—and who are now struggling to pay for ARVs?

Most of these patients have made great sacrifices to access ARV treatment. They have kept appointments at the clinic, and they have been adherent to their treatment regimen as long as money was found to purchase ARVs. In contrast, other individuals may have delayed being tested for HIV, while continuing risky behaviour, and only accepted HIV testing because of the promise of free ARVs. Many of them have not been as ill as those who have managed to somehow find resources to initiate treatment.

Since the increased access to free ARVs, a sharp increase in HIV testing has been noted at voluntary testing and counselling sites in Kampala, Uganda, and also in many other countries [11]. In Uganda in the Mulago–Mbarara Teaching Hospitals' Joint AIDS Programme supported by the President's Emergency Plan for AIDS Relief, 15,000 patients were tested for HIV in the last seven months, and 40% of these patients were HIV positive. Although these newly diagnosed ARV-naive patients who meet the biological criteria for ARV treatment should be treated, we must be able to also prioritise individuals for ARVs who are “HIV veterans” and are now barely finding money or have already exhausted their resources to pay for their drugs.

Today in Uganda, ARVs provided by the Multi-Country AIDS Programme, the Global Fund to Fight AIDS, Tuberculosis, and Malaria, and the President's Emergency Plan for AIDS Relief are also used for patients that previously paid for the drugs themselves. Nevertheless, many patients still have not been taken in by these free ARV programmes. It is important that ARV roll-out projects in other countries learn from this experience in Uganda.

## Abbreviation

ARV: antiretroviral
